# Does facial soft tissue protect against zygomatic fractures? Results of a finite element analysis

**DOI:** 10.1186/s13005-015-0078-5

**Published:** 2015-06-16

**Authors:** Heike Huempfner-Hierl, Alexander Bohne, Andreas Schaller, Gert Wollny, Thomas Hierl

**Affiliations:** Department of Oral & Maxillofacial Plastic Surgery, Leipzig University, Liebigstrasse 12, 04103 Leipzig, Germany; BBG Bodenbearbeitungsgeraete Leipzig GmbH & Co KG, Leipzig, Germany; Biomedical Imaging Technologies, ETSI Telecomunicación, Universidad Politécnica de Madrid, Ciudad Universitaria s/n, 28040 Madrid, Spain

**Keywords:** Biomechanics, Zygomatic fracture, Facial soft tissue, Finite element analysis

## Abstract

**Introduction:**

Zygomatic fractures form a major entity in craniomaxillofacial traumatology. Few studies have dealt with biomechanical basics and none with the role of the facial soft tissues. Therefore this study should investigate, whether facial soft tissue plays a protecting role in lateral midfacial trauma.

**Methods:**

A head-to-head encounter was simulated by way of finite element analysis. In two scenarios this impact - with and without soft tissues - was investigated to demonstrate the potential protective effects. To achieve realism, a transient simulation was chosen, which considers temporal dynamics and realistic material parameters derived from CT grey values.

**Results:**

The simulation results presented a typical zygomatic fracture with all relevant fracture lines. Including soft tissues did not change the maximum bony stress pattern, but increased the time period from impact to maximal stresses by 1.3 msec.

**Conclusions:**

Although this could have clinical implications, facial soft tissues may be disregarded in biomechanical simulations of the lateral midface, if only the bony structures are to be investigated. Soft tissue seems to act as a temporal buffer only.

## Introduction

Lateral midfacial zygomatic fractures are frequently encountered in craniomaxillofacial traumatology. Typical causes are assaults, traffic accidents, or sports incidents [1–3]. Here a frequent situation is a player versus player impact in team sports like association football or rugby. Depending on local cultural habits 13 to 30 % of all sport-sustained fractures in the head and neck area are located in the lateral midface [2–7]. Typical victims are males aged between 18 to the mid thirties. The causative blunt impact often results from a head-to-head encounter as two players try to hit the ball with their heads, one reaching the ball, the other one his opponent’s zygoma.

Concerning biomechanical studies about facial traumatology researchers will always be confronted with certain difficulties. Many experiments have been performed on cadavers. Evidently, only restricted conclusions can be made, as cadavers will have undergone postmortal alterations and, in most cases, will not have been of the typical age group of persons suffering from zygomatic fractures. Moreover cadaver specimen will be destroyed in these experiment so that they are not repeatable.

Attempts have been made with small and big animal models, but whereas the anatomy of a sheep tibia may be comparable to the human tibia in a certain extent [8], the human facial skull will not be really represented by any animal model.

Since about thirty years finite element analysis (FEA) has expanded from technical application into biomechanical and medical research. Finite element models (FE-models) have developed from rather simple models at the beginning to very sophisticated 3D-models with increasing computing capacity and improving methods of data acquisition [9–12].

The authors have shown that finite element analysis can reproduce a head collision leading to a typical fracture pattern in a previous study without the integration of midfacial soft tissue [12]. Regarding further biomechanical literature on zygomatic trauma, only reports concentrating on the field of zygomatic fracture osteosynthesis or necessary impact forces have been published by now [13–15].

Published studies investigating adjacent anatomical regions like the orbit or maxilla concentrated on bone stresses and have neglected facial soft tissue in their simulations [9–12, 15].

The question arises, whether this simplification is acceptable, and how simulation of biomechanical parameters of facial soft tissue and bone would alter fracture patterns and stress propagation in the simulation of zygomatic fractures.

To answer this question a biomechanical study based on finite element analysis was initiated to investigate the influence of facial soft tissue in protecting against zygomatic fracture. The null hypothesis was that the facial soft tissue envelope would protect the lateral midface and would change the fracture pattern in a typical head-to-head encounter.

## Methods

Two scenarios of head-to-head impacts as forehead versus zygoma impacts were created in ANSYS Workbench (ANSYS Classic V12.0.1; ANSYS Inc. Canonsburg, PA, USA). The first consisted of finite element models of two skulls without any soft tissue whereas in the second scenario soft tissue parameters were included in the victim’s skull model. Besides presence of soft tissue, all other parameters were identical.

### Model construction

**Fig. 1:**
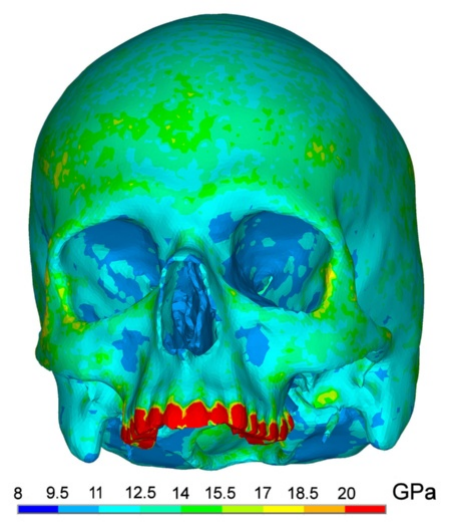
Individual material parameters of the skull model as calculated with BoneMat. Young’s moduli are given in gigapascal [GPa]. Stronger bone is found in the orbital rim region, zygomatic body and paranasal/zygomatic buttresses

For creating the finite element models of victim and assailant a CT scan of a young healthy non-obese white male individual without any pathological structures or previous surgery was chosen (1 mm contiguous slicing, Siemens Volume Zoom Plus, Siemens Germany). The CT scan was segmented in Vworks 4.0^Surgery^ (Cybermed Co., Seoul, Korea). In the first step a threshold-based segmentation was performed to distinguish between bone and non-bone structures. Then each slice was manually edited to erase artefacts and add missing thin cortical structures, e.g. within the orbital walls. The resulting skull was exported in STL format and imported into ANSYS ICEM CFD 12.0.1. Here a finite element volume mesh consisting of 736 934 10-node tetrahedrons was created. To increase realism of the victim’s skull no uniform material parameters were used. Instead they were refined by attributing computed individual material values. Therefore Young’s moduli of each individual element of the victims skull were calculated according to the respective grey value of the CT scan (Hounsfield unit). This was accomplished by using the programme BoneMat® developed by Taddei et al. [16] (Fig. 1) and the proceeding suggested by Morgan et al. [17]. There was no specific modelling of bony sutures, only their differing grey scale values were considered. Poisson ratio and density were defined as 0.326 [18] and 1.591 g/cm^3^ [19]. For the impacting skull a uniform Young’s modulus of 13 500 Megapascal (MPa) was chosen to keep calculation effort reasonable [11, 19].

### Impact scenario

**Fig. 2:**
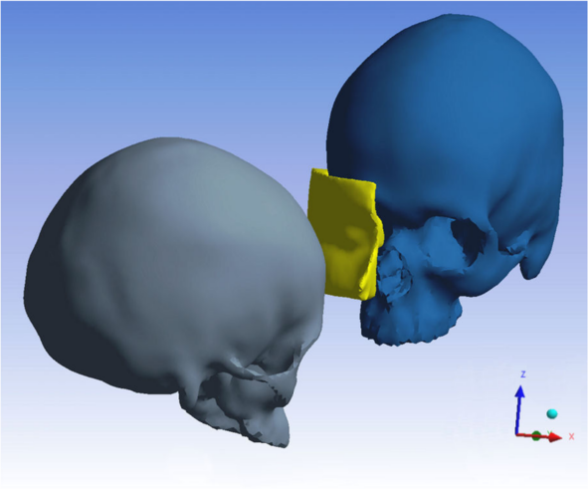
Experimental set-up of the headbutt including the facial soft tissue of the victim. The impacting skull is inclined 15°caudally to the Frankfort Horizontal and hits with the forehead

As a typical sports accident was to be created, an impact with a running assailant was assumed with 6.5 m/s velocity [20]. The head butt was performed with 15° caudal inclination of the impacting skull hitting the zygomatic prominence of the victim (Fig. 2). A transient nonlinear solution was chosen because of the time dependency of the applied force and the impactor-bone interaction. Especially for a fast phenomenon like depicted here the transient approach, which implies a gradient oscillation-like excitation of the struck skull, seemed appropriate.

### Soft tissue simulation

**Fig. 3:**
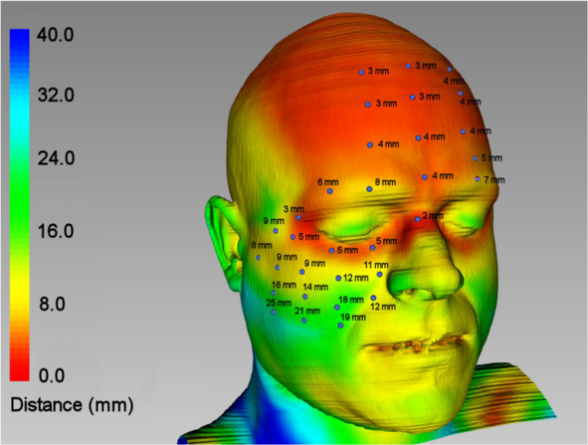
Soft tissue thickness map based on CT measurements. All values are rounded. Values lie between 3 to 4 mm in the forehead, whereas up to 25 mm are reached in the cheek region

For calculating the facial soft tissue simplifications were made. First, homogenous material parameters were used for all soft tissues instead of distinguishing between skin, muscle, and fat. A Young’s modulus of 0.5 MPa, density of 1.1 g/cm^3^ and Poisson ratio of 0.45 [21, 22] were attributed, creating a model of 152 765 elements. Second, the soft tissue coverage of the impacting forehead was omitted. As it consists of a uniform thin layer of skin and muscle of three to four millimetres (Fig. 3), which is less compressible than the much thicker soft tissue of the cheek, this layer was not incorporated. To calculate soft tissue thickness the CT scan was segmented for bone and soft tissue and the distances between both surfaces were computed by defining the shortest distance from outer surface to bone. For this Facial Analysis Tool (FAT) was utilized, a VTK (Visualization Toolkit, Kitware, Clifton Park, USA) based programme, which has been developed at the authors’ institution [23]. So a soft tissue area covering the impact region in size of 19.7 cm for the width and 13.5 cm for the height was integrated into FEA-simulation.

### Boundary conditions

To achieve a realistic numerical calculation of stresses, especially at the bone surface, the Young’s modulus of the skull was modified by limiting the lowest value to 11 000 MPa according to experimental studies [11, 19]. Concerning boundary conditions, the nodes of the occipital condyles of the victims’ skull were fixed in all degrees of freedom. A coulomb friction model was created in which the solid body friction was divided into sticking and sliding friction and a coefficient of 0.4 was assigned [24]. The assaulting head was not fixed, but velocity and vector were defined. For evaluation both scenarios were analyzed regarding the time-dependent propagation of stresses within soft tissue and bone. Moreover the final stress patterns were compared.

Von Mises stresses were evaluated for both scenarios. According to the studies of Nagasao et al. [11] a yield criterion of 153 MPa was defined, where material parameters change from elastic to plastic behaviour. Higher values will cause fractures represented by plastic material deformation. For the analysis of potential fractures a theory of mechanical engineering was utilized, which states that failure of the examined part will occur, if two stress gradients meet above the yield limit. This represents the point, at which the object will fail to resist the load and break [25–27].

It is widely approved that this corresponds with fractures of facial bone [11, 28].

According to the statutes of the local ethical review committee, no approval of this study had been necessary.

## Results

**Fig. 4:**
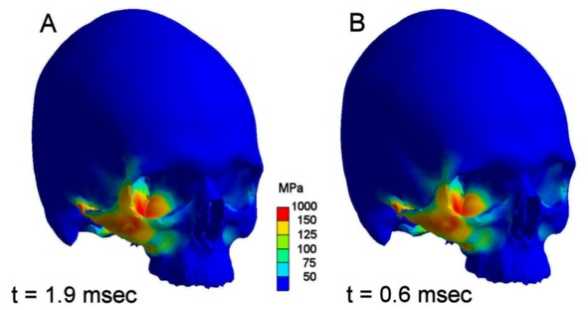
Comparison of maximum stress pattern in both scenarios. Left (**a**) with, right (**b**) without soft tissue simulation. Regions coloured red are above the assumed failure of bone (150 MPa) and represent a typical zygomatic fracture. The zygomatic arch shows also high stresses. This maximum stress pattern will be reached 0.6 (**b**) respectively 1.9 msec (**a**) after impact

**Fig. 5:**
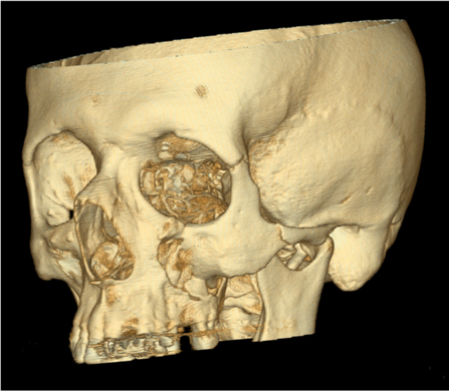
Typical CT scan presenting all typical fracture-related details. Comparison to Fig. 4 shows the close resemblance to the simulation

Finite element analysis using a highly detailed dense volume mesh consisting of 736,934 elements and a mode of transient simulation revealed a complex stress pattern with almost identical distribution of maximum stresses in both scenarios. Major stresses could be noted in the impact area. Anatomical borders of stresses beyond the yield criterion of bone were the zygomatic crista, the lateral orbital rim, the orbital floor, the infraorbital rim, and the zygomatic arch. From a clinical viewpoint this equals a typical lateral midfacial zygomatic fracture (Figs. 4, 5).

**Fig. 6:**
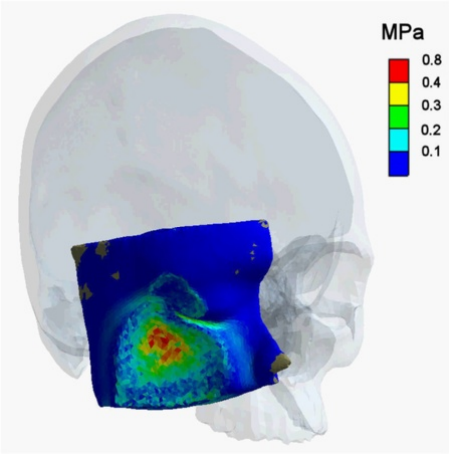
Soft tissue stresses. Highest values are seen in the direct contact zone, especially at the perimeter of the impacting forehead (red values)

Regarding stresses arising in soft tissue, up to 0.77 MPa were seen in the contact zone. Highest values could be found at the perimeter surrounding the impacting forehead area (Fig. 6).

**Fig. 7:**
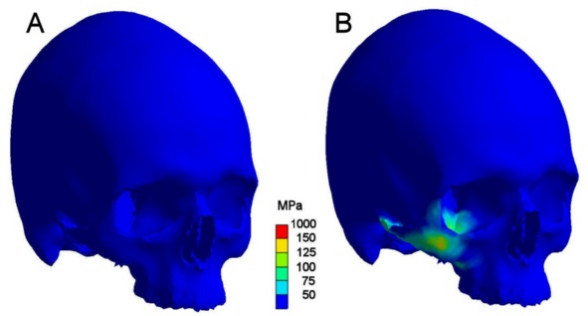
Situation at 0.15 msec after impact. **a**: (left skull) scenario with soft tissue simulation; **b**: (right skull) scenario without soft tissue. Stresses are displayed in megapascal [MPa]. Without facial soft tissue coverage simulation stresses are propagating within the zygoma, whereas no stresses are present in bone with soft tissue simulation at that time

**Fig. 8:**
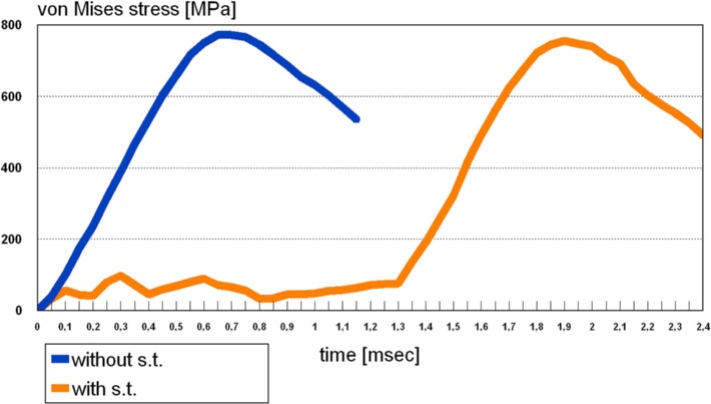
Comparison of stress curves in the simulation without and with soft tissue (s. t.). The temporal delay caused by simulation of soft tissue is clearly recognizable

**Fig. 9:**
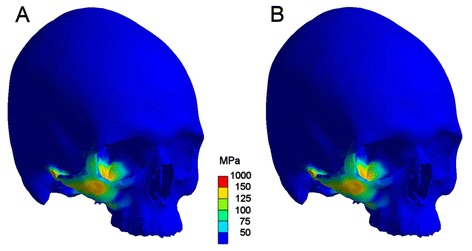
Intermediate stress situation. **a**: (left) scenario with included soft tissue at 1.5 msec after impact; **b**: (right) scenario without soft tissue at timepoint 0.24 msec after impact. An identical stress pattern is displayed, the only difference is the time shift of 1.26 msec due to the buffer effect of the midfacial soft tissue envelope

The second question was, whether any differences regarding the time dependency of stress propagation could be seen, i.e. whether the facial soft tissues would act as a kind of buffer between the two skulls. Therefore both trauma scenarios were compared on a time line ranging from the start of the impact until 2.4 msec, when the full stress propagation had been reached in the model including soft tissue. As in the set-up without soft tissue simulation the full stress pattern was reached 0.6 msec after impact, the soft tissue buffer increased this period to 1.9 msec. Comparing the scenarios, no bone stresses could be seen in the soft tissue model up to 1.3 msec, whereas at 0.15 msec a first loading of the zygomatic body was present in the pure bone scenario (Fig. 7). At 0.6 msec the maximum stress pattern occurred in the bone model, whereas no loading could be noticed in the bone of soft tissue scenario (Fig. 8). An intermediate stress situation in the zygoma was apparent at 0.24 msec in the scenario without soft tissue representation respectively at 1.5 msec with soft tissue (Fig. 9).

Finally, the maximum stress distribution was reached at 1.9 msec in the soft tissue set-up with an identical pattern as in the bone model (Fig. 8). In this specific set-up critical values above the yield criterion could be seen on the impact side. Lower, uncritical stresses were present on the opposite side in the region of the zygomatic buttress, the orbital floor, and lateral orbital rim. In both scenarios, a peak impact force of 45 N at the impact site could be determined.

So in both FEA-simulations – with and without bone covering facial soft tissue – the same maximum stress pattern had to be registered. In analysing time dependency a delay of 1.3 msec for reaching the full stress pattern had been found. Comparison of stress curves as a function of time are displayed in Fig. 8. Here it is easily discernable that in the model with simulation of soft tissue bone stresses will occur at a time, when they will already have finished in the bone only model. But the resulting maximum von Mises stresses are almost identical.

## Discussion

In a biomechanical investigation based on finite element analysis the role of the facial soft tissue in blunt zygomatic trauma was investigated. In two scenarios a head-to-head encounter like seen in sports’ accidents was modelled, one with and the other one without simulating the soft tissue coverage. The results showed that the midfacial soft tissue led to a delay of impact stresses of 1.3 msec compared to the pure bone scenario. Soft tissue did not change the peak stresses or the slope of stress build-up. So the postulated null hypothesis which supposed a protective effect of the soft tissue envelope in this trauma scenario was rejected.

The following aspects are of interest for the final analysis: the tool chain in itself, the computational results, and the influence of the modeling of soft tissue for the obtained results.

The tool chain permitted the creation of highly detailed bone volume meshes and allowed the use of individual material parameters for the investigated bone. As explained in previous studies [12, 21] and by Szwedowski et al. [29] using patient-specific Young’s moduli for bone derived from the individual CT-scan should increase the quality of the study and is a step towards the individualization of craniofacial biomechanical simulation.

However, regarding the soft tissue two simplifications were made: First, the scalp of the impacting head was not modeled because the scalp is a uniform thin layer that is hardly compressible in contrast to the three-to-sevenfold thicker and more pliable cheek. Our measurement of 3 to 4 mm scalp thickness derived from an automatic software-based calculation corresponds well to data taken from literature and measurements performed on the CT-scan [30, 31]. Additionally, facial soft tissues were modelled as one material and was not divided into skin, fat, and muscle. This seems reasonable according to the work of Zachow et al. [22], who reported on the use of uniform material parameters for maxillofacial surgery simulation. In literature, varying material parameters for Young’s moduli and Poisson ratio have been reported for soft tissue. In a survey Choi and Zheng [32] stated values for Poisson ratios from 0.3 to 0.5. Here the suggestions of Zachow et al. [22] were followed, who suggested a value between 0.43 and 0.45. Regarding material parameters soft tissue shows the peculiarity that they depend on the extent of deformation [32]. In our study a Young’s modulus of 0.5 MPa was chosen, which lies in the range of the results of Choi and Zheng, who found 0.59 to 0.6 MPa, and Zachow et al. [22]. Two further conditions of the scenario have to be mentioned. First, the impact was modelled as a head butt without full body impact like seen in American football, in which full body mass instead of the head mass should be taken for the encounter [33]. Secondly, neck flexion in impactor and victim as well as hair were not considered. Modelling neck flexion is important, if neck strain is to be evaluated, but as this was not intended in this investigation, it was omitted.

The second point of discussion is the accordance of the simulated fracture pattern with clinical findings. Here the model simulates all relevant fracture lines ranging from the lateral orbital wall, infraorbital rim, zygomatic buttress, and zygomatic arch. Even potential comminution of the zygomatic buttress is resembled and equals typical clinical CT scans (Fig. 5). Thus it can be stated that our model created a clinically correct copy of a typical lateral midfacial fracture.

The last issue is the influence of facial soft tissue regarding a blunt impact, in this specific case a head-butt as might be encountered in a sports event. The resulting stress patterns were almost identical for the chosen set-up and equalled a typical zygomatic fracture. Thus, under our chosen model specifications, facial soft tissue would not change the resulting fracture type. Whether there could be changes regarding the amount of comminution cannot be judged from our results. The question remains why no decrease in peak stresses could by seen in the soft tissue scenario. A possible answer could lie in the fact that the cheek is pliable and shows a lateral displacement if hit by a blunt impactor. As there are no obstacles for this soft tissue shift, the impactor would hit the remaining thin layer of skin and remaining fat after 1.3 msec delay. It can be concluded by the results that this lateral soft tissue displacement would not decrease the impact force substantially as identical peak stress values and slope of stress build-up were encountered in the zygoma.

As demonstrated, soft tissue seems to act as a temporal buffer increasing time period to maximum stresses in bone from 0.6 to 1.9 msec. This delay of 1.3 msec could have clinical implications as soft tissue could decrease the impact and consecutive acceleration of the concomitant brain tissues. As investigations on brain trauma biomechanics focus on linear and angular acceleration and not on impact force the effect can not be calculated [34, 35]. Therefore the potential effect of reducing the probability of subdural hematoma via bridge vein rupture or focal brain concussion will not be discussed here. A further possible effect of impact delay could lie in allowing the victim to evade from the impactor or perform defensive moves.

On the other hand it is questionable, whether a delay of only 1.3 msec for reaching the full stress pattern in the soft tissue scenario in comparison to the bone only scenario will make any noteworthy difference concerning the clinical result.

It is difficult to discuss our findings with preexisting literature as this investigation is according to our knowledge the first study on the influence of soft tissue on craniomaxillofacial trauma patterns. The only result which may be sufficiently discussed is the question whether the resulting stress pattern resembles a typical zygomatic fracture. As mentioned above the pattern displayed in Fig. 4 is in accordance with the findings demonstrated in existing studies [36, 37].

## Conclusion

Within the limitations of this study, it can be concluded that facial soft tissue has only little influence on maximum stresses in bone and fracture pattern in case of a blunt head-to-head encounter.

The simulation of a head impact to the lateral midface with simulation of soft tissue results in protracting the maximum stress pattern by 1.3 msec.

Whether this acts as buffer cannot really be answered. Our results suggest that facial soft tissue does not really play a decisive role for protecting the facial skull.

Furthermore they suggest that soft tissue simulation may be disregarded in the lateral midface in instances, in which only stress patterns would be examined as it complicates simulation. So the results concerning influence of soft tissue support and justify existing FEA-models, in which soft tissue is neglected.

Facial soft tissue simulation will be a necessary step in special clinical questions like the effects of protective devices in sports which will be addressed in future investigations. The results demonstrate that finite element simulation is an appropriate means to perform biomechanical investigations as they correlate well with clinical findings.
